# Supplementary material for: Preparation and Characterization of Fe-Mn Binary Oxide/Mulberry Stem Biochar Composite Adsorbent and Adsorption of Cr(VI) from Aqueous Solution
Source: Int J Environ Res Public Health. 2020 Jan 21;17(3):676. doi: 10.3390/ijerph17030676 (PMC7036895; doi:10.3390/ijerph17030676)
Supplement: Supplementary file 1 [file ijerph-17-00676-s001.pdf]

Supporting Information

Preparation and characterization of Fe-Mn binary oxide/mulberry stem biochar composite adsorbent and its adsorption of Cr(VI) from aqueous solution

Meina Liang[1,2,*], Shuiping Xu[1], Yinian Zhu[1,2,*], Xu Chen[1], Zhenliang Deng[1], Liling Yan[1], Huijun He[1,2]

Affiliations:

[1] College of Environmental Science and Engineering, Guilin University of Technology, Guilin, Guangxi 541004, P.R. China

[2] Guangxi Key Laboratory of Environmental Pollution Control Theory and Technology, Guilin, Guangxi 541004, P.R. China

* Correspondence author.

E-mail address: liangmeinaa@163.com, 15078367985; zhuyinian@glut.edu.cn, 13077692937.

S1:

Table 1. Composition analysis of MBC and FM-MBC before and after Cr(VI) adsorption.

Absorbent	Atomic content (%)				
	C	H	N	O	S
MBC	77.4	2.31	0.47	8.94	0.88
FM-MBC	57.29	2.52	1.02	28.31	0.83

S2:

Mineral elements

The elemental composition of MBC (Table 2) contained a large amount of Ca, K and Mg, and trace amounts of minerals such as Al, Fe and Mn. The pyrolysis process concentrated and enriched these components, which mainly existed in the form of ash. After modification, Ca and K contents in FM-MBC decreased whilst Fe, Mn and Mg increased (0.079 to 9.610 %, 0.003 to 2.031 % and 0.435 to 0.681 %, respectively). As the C content of the modified biochar decreased, therefore Fe, Mn and Mg oxides occupied C on the surface of the biochar.

Table 2 Mineral element analysis of MBC and FM-MBC

	K	Ca	Mg	Cu	Al	Zn	Ni	Fe	Mn
MBC (%)	1.264	3.172	0.435	0.001	0.109	0.127	0.001	0.079	0.003
FM-MBC (%)	0.120	0.937	0.681	0.001	0.000	0.030	0.000	9.610	2.031

S3:

Zeta potential

The results of the zeta potential at different pH levels (Fig. 1) indicated that the zeta potential of FM-MBC was equal to 0 when pH was approximately equal to 7.4, indicating that the pH_{PZC} of FM-MBC was equal to 7.4. Previous results by Zhang et al.[1] (Zhang et al., 2015) showed that when $pH < pH_{zpc}$, the surface of the biochar was protonated and positively charged. Under the action of electrostatic attraction, Cr-containing anion migrated to the surface of the positively charged biochar, thereby increasing the adsorption capacity of the biochar. Chen et al. [2](Chen et al., 2018) showed that the higher the pH_{zpc}

value, the greater is the possibility that the adsorbent will be positively charged in a solution with a wide range of pH values, thereby facilitating the adsorption of anions by electrostatic interaction.

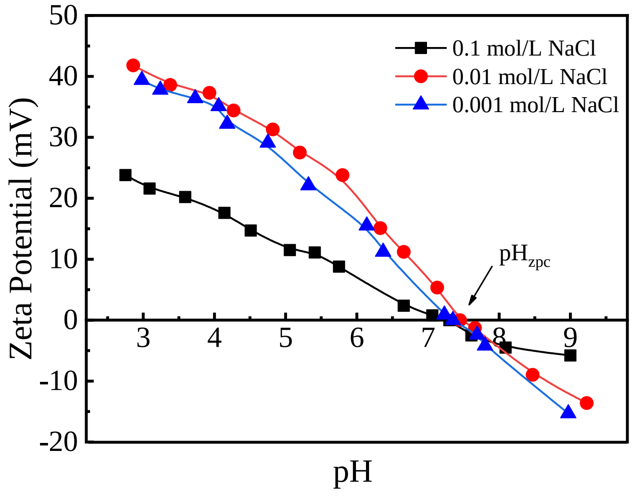

Figure S1 Zeta potential of FM-MBC as function of pH

S5:

Table 3. XPS peak spectral analysis of FM-MBC and FM-MBC after Cr(VI) adsorption.

Sample	Bonding energy (eV)					Composition (%)				
	C1s	N1s	Fe2p	Mn2p	Cr2p	C1s	N1s	Fe2p	Mn2p	Cr2p
FM-MBC	284.42	399.69	710.55	640.48	-	66.32	2.96	4.62	1.03	-
FM-MBC after Cr(VI) adsorption	284.35	399.94	710.76	641.03	576.72	67.26	1.3	3.94	0.43	2.06

References

1. Zhang M.M.; Liu Y.G.; Li T.T.; Xu W.H.; Zheng B.H.; Tan X.F.; Wang H.; Guo Y.M.; Guo F.Y.; Wang S.F. Chitosan modification of magnetic biochar produced from Eichhornia crassipes for enhanced sorption of Cr(VI) from aqueous solution. *RSC. Advances.* **2015**, *5(58)*, 46955-46964.

2. Chen Y.Y.; Wang B.Y., Xin J., Sun P., Wu D.; 2018. Adsorption behavior and mechanism of Cr(VI) by modified biochar derived from Enteromorpha prolifera. *Ecotoxicol. Environ. Saf.* **2018** *164*, 440-447.
